# Supplementary material for: Targeting of SLC25A22 boosts the immunotherapeutic response in KRAS-mutant colorectal cancer
Source: Nat Commun. 2023 Aug 4;14:4677. doi: 10.1038/s41467-023-39571-6 (PMC10403583; doi:10.1038/s41467-023-39571-6)
Supplement: Supplementary file 1 — Supplementary Information [file 41467_2023_39571_MOESM1_ESM.pdf]

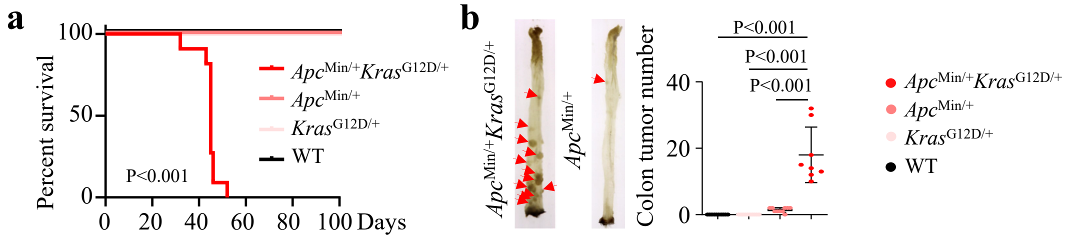

Fig. S1. Apc^{Min} and Kras^{G12D} promoted colonic tumorigenesis in mice. (a) Apc$^{Min/+}$Kras$^{G12D/+}$Villin-Cre mice (n=8) had reduced survival and **(b)** increased tumorigenesis compared to wildtype (n=10), Apc$^{Min/+}$ (n=10) and Kras$^{G12D/+}$Villin-Cre mice (n=10). Data are presented as means ± SEM for growth curve **(a)** and ± SD for tumor number **(b)**. Two-tailed two-way ANOVA for growth curve comparison **(a)**. Two-tailed one-way ANOVA **(b)**.

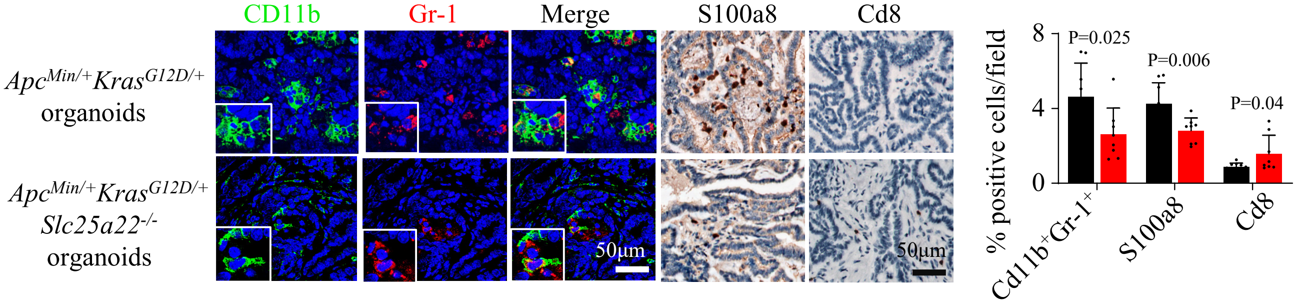

Fig. S2. Immune cell infiltration in the APC-KRAS organoid allograft model. Immunofluorescence staining and immunohistochemistry of markers of MDSC and CD8^{+} T-cells in APC-KRAS organoid allografts (n=4). Data are presented as mean ± SD. Each dot represented the value from one capture field. Two-tailed Student's t-test. Source data are provided as a Source Data file.

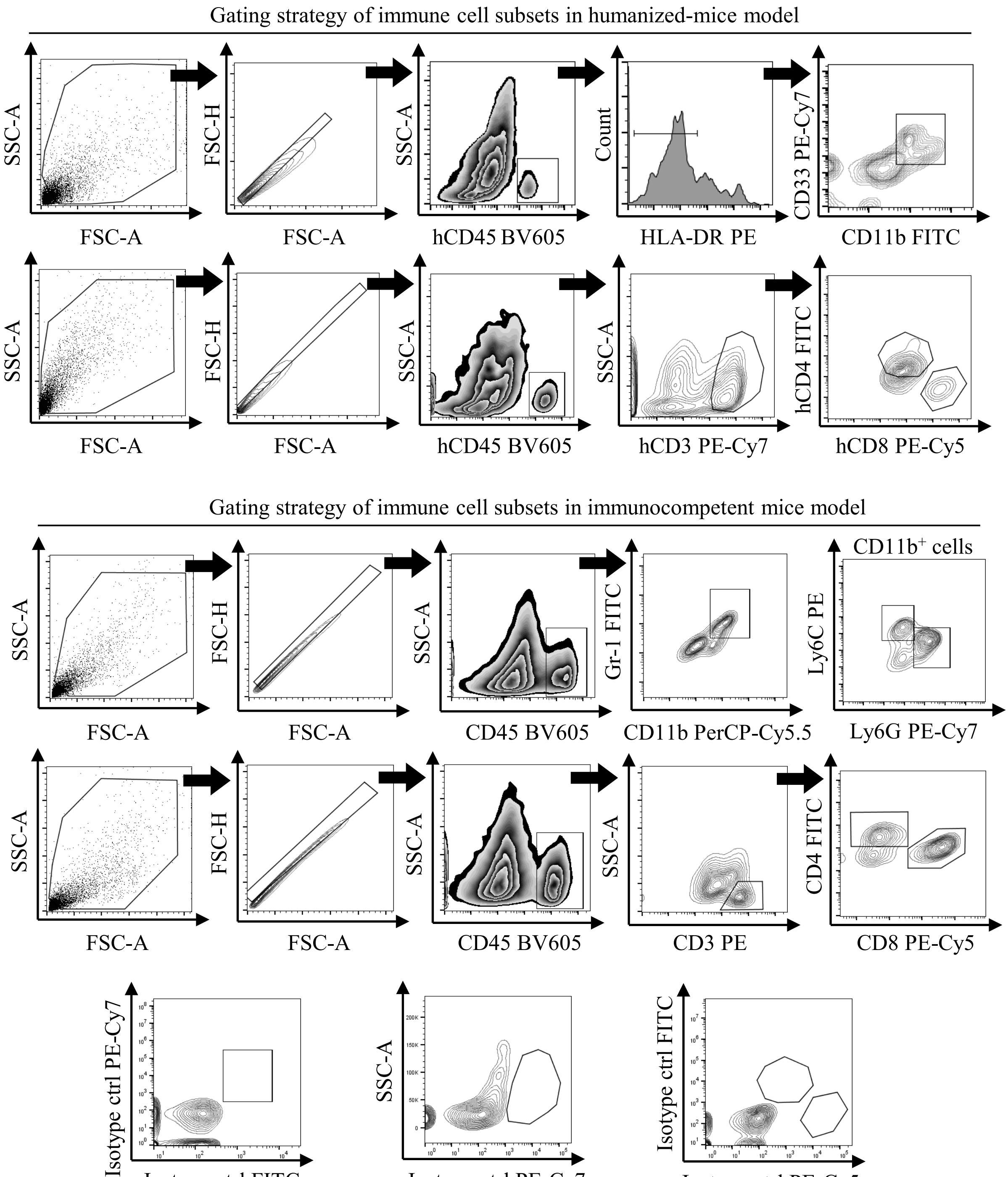

Fig. S3. Gating strategy to identity the immune cell subsets in humanized and immunocompetent mice by flow cytometry.

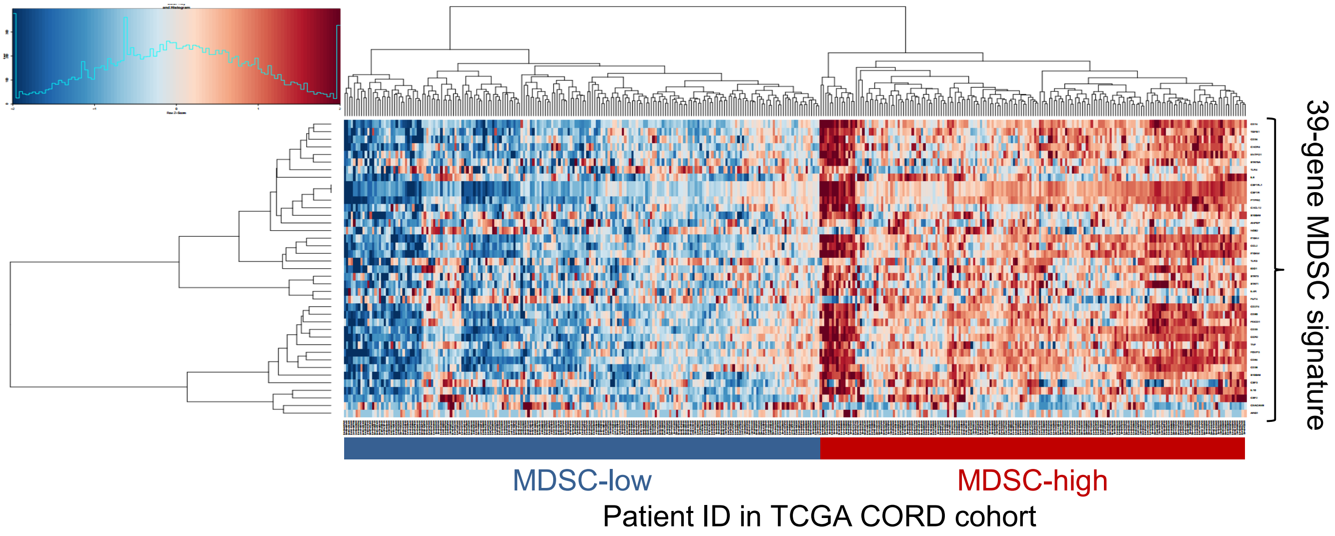

Fig. S4. Analysis of MDSC infiltration based on RNA-seq dataset from TCGA. TCGA CRC RNA-seq cohort was divided into MDSC-high (n=181) and MDSC-low CRC (n=202) based on the expression of 39 signature genes. Source data are provided as a Source Data file.

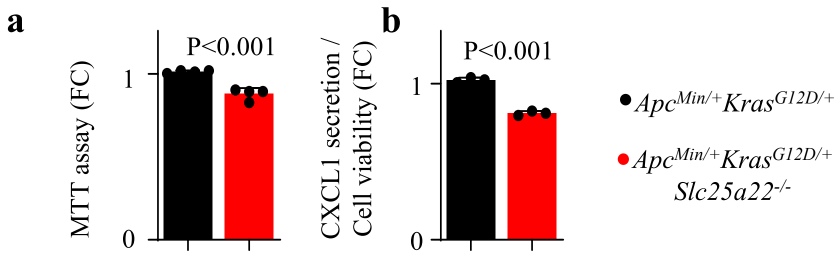

Fig. S5. Effect of SLC25A22 knockout on cell viability and CXCL1 in APC-KRAS organoids. (a) SLC25A22 knockout suppressed organoid growth (n=4). **(b)** SLC25A22 reduced CXCL1 secretion after normalization by cell viability (n=3). Each dot represents an independent sample. Data are presented as mean ± SD **(a, b).** Two-tailed Student's t-test **(a, b)**. Source data are provided as a Source Data file.

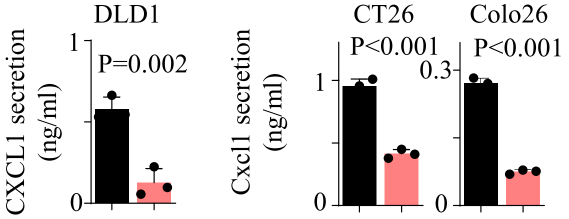

Fig. S6. CXCL1 in KRAS-mutant CRC cells. Validation of CXCL1 knockdown in DLD1 (n=3), CT26 (n=3) and Colo26 cells (n=3) by ELISA. Each dot represents an independent sample. Data are presented as mean ± SD. Two-tailed Student's t-test. Source data are provided as a Source Data file.

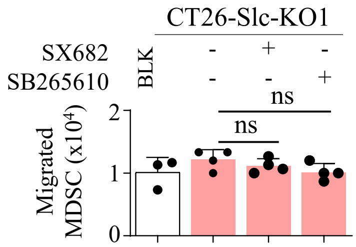

Fig. S7. Effect of CXCR2 inhibiters on MDSC migration. Co-incubation with SX682 (n=4) or SB265610 (n=4) with SLC25A22 knockout cells conditioned medium had no significant effect on MSDC migration in transwell assay. Each dot represents an independent sample. Data are presented as mean ± SD. Two-tailed Student's t-test for two group comparison. ns, no significance. Source data are provided as a Source Data file.

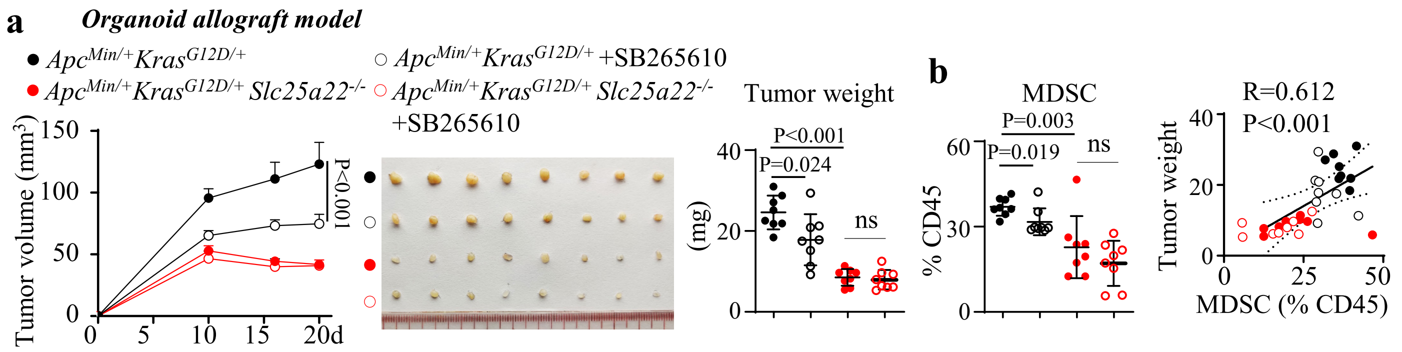

Fig. S8. SLC25A22-mediated MDSC recruitment promotes KRAS-mutant CRC organoid allograft growth. (a) SB265610 inhibited growth of Apc$^{Min/+}$Kras$^{G12D/+}$ organoid allografts, but had no effect on Apc$^{Min/+}$Kras$^{G12D/+}$Slc25a22$^{-/-}$ organoid allografts in C57BL/6 mice (n=8). **(b)** SB265610 down-regulated MDSC in Apc$^{Min/+}$Kras$^{G12D/+}$ organoid allografts. Tumoral MDSC positively correlated with tumor weight (n=8). Each dot represents an independent tumor. Data are presented as mean ± SEM for growth curve **(A)** and ± SD **(a, b)**. Two-tailed two-way ANOVA for growth curve comparison **(a)**. Two-tailed Student's t-test for two group comparison **(a, b).** Two-tailed Pearson correlation test **(b)**. ns, no significance. Source data are provided as a Source Data file.

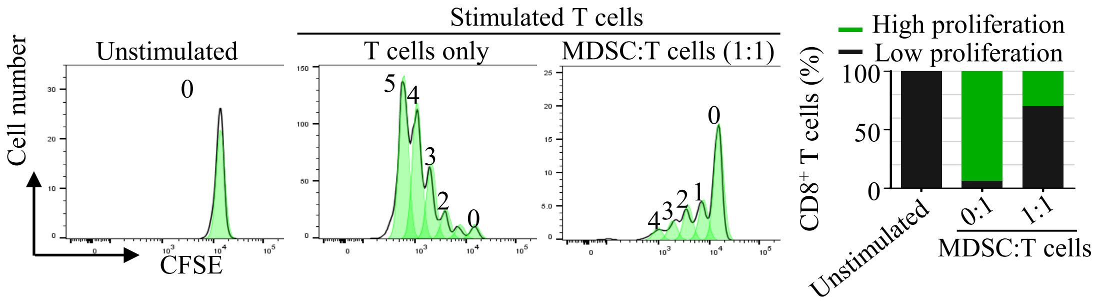

Fig. S9. MDSC suppressed T cell proliferation in vitro. Naïve T cells were harvested from mouse spleen and loaded with carboxyfluorescein diacetate (CFSE) fluorescence dye. MDSC and T cells are co-cultured in vitro in the presence of Dynabeads Mouse T-Activator CD3/CD28 (n=3). The number of proliferative cycles was determined by FlowJo software. Source data are provided as a Source Data file.

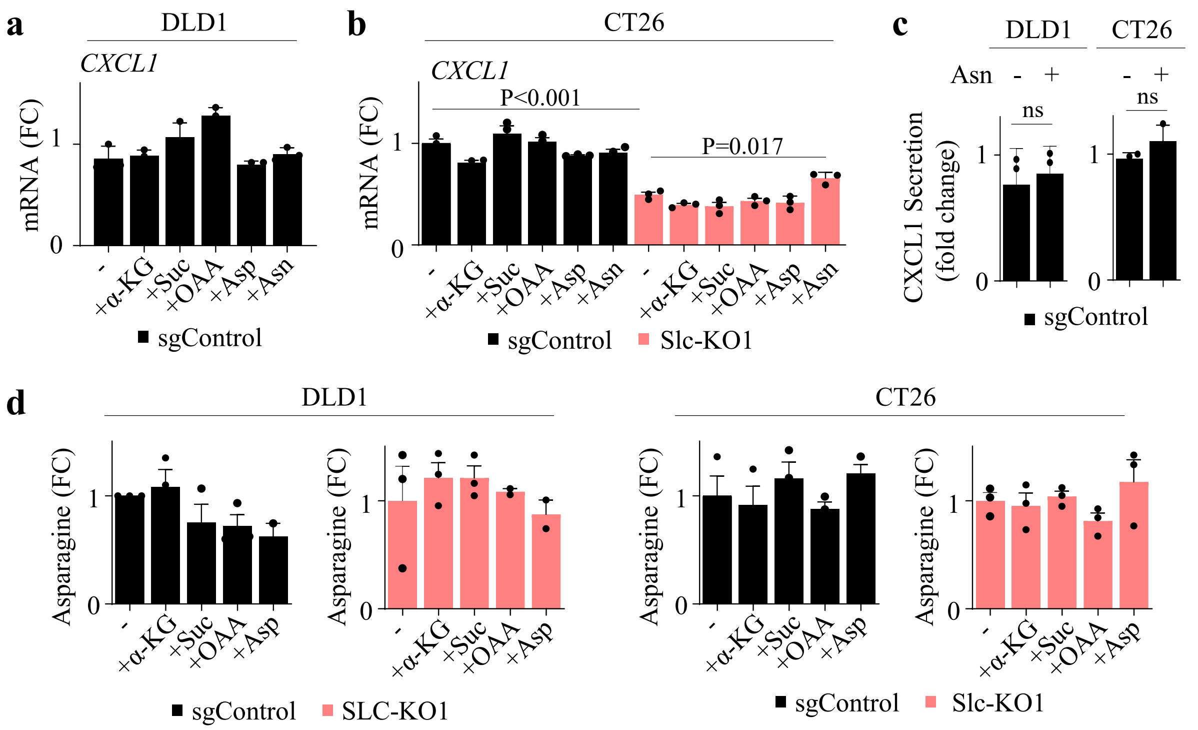

Fig. S10. Effect of metabolites on CXCL1 and asparagine. (a) Metabolites addition (2mM, 24h) had no effect on CXCL1 mRNA in DLD1-sgControl cells (n=3). **(b)** Asparagine (2mM, 24h) restored CXCL1 mRNA in CT26-Slc-KO cells, but had no effect on sgControl cells (n=3). **(c)** Asparagine (2mM, 24h) had no effect on secretion of CXCL1 in DLD1-sgControl and CT26-sgControl cells (n=3). **(d)** Addition of TCA cycle metabolites or aspartate (2mM, 24h) had no effect on intracellular levels of asparagine in DLD1 and CT26 cells (n=3). Each dot represents an independent sample. Data are presented as mean ± SD **(a-d).** Two-tailed Student's t-test for two group comparison **(a-d).** ns, no significance. Source data are provided as a Source Data file.

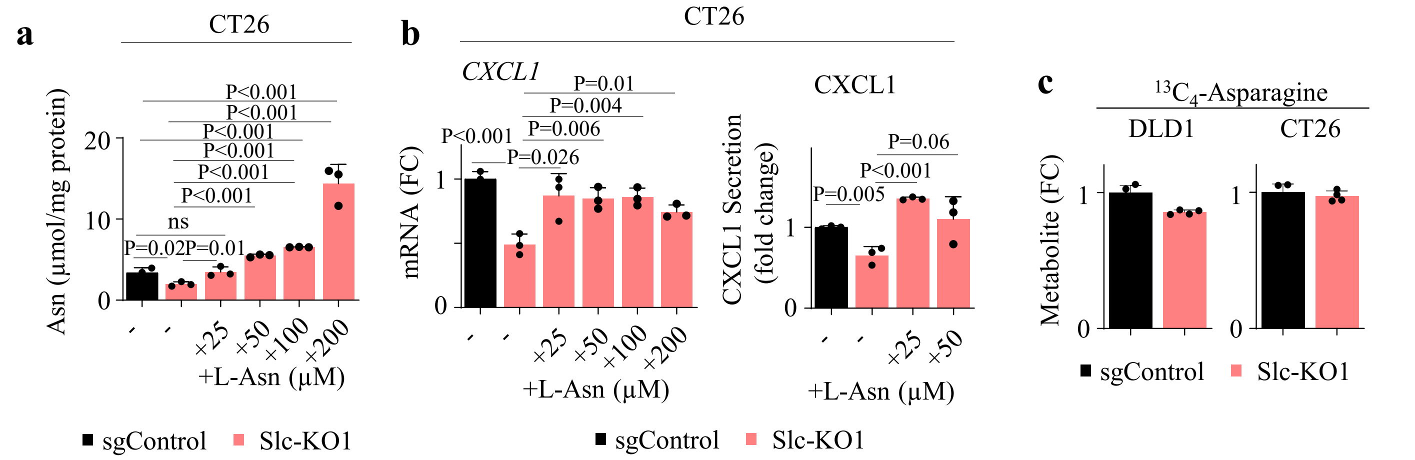

Fig. S11. Low-dose asparagine restored CXCL1 expression. (a) Titration of the asparagine dose required to restore intracellular asparagine in CT26 cells (n=3). **(b)** Low-dose asparagine (25μM, 24h) restored CXCL1 expression and secretion in CT26-Slc-KO cells (n=3). **(c)** Cells were treated with $^{13}C_4$-Aspartate (2mM, 96h) and their conversion to $^{13}C_4$-Asparagine was determined by LC-MS (n=4). Each dot represents an independent sample. Data are presented as mean ± SD **(a-c).** Two-tailed Student's t-test for two group comparison **(a-c)**. ns, no significance. Source data are provided as a Source Data file.

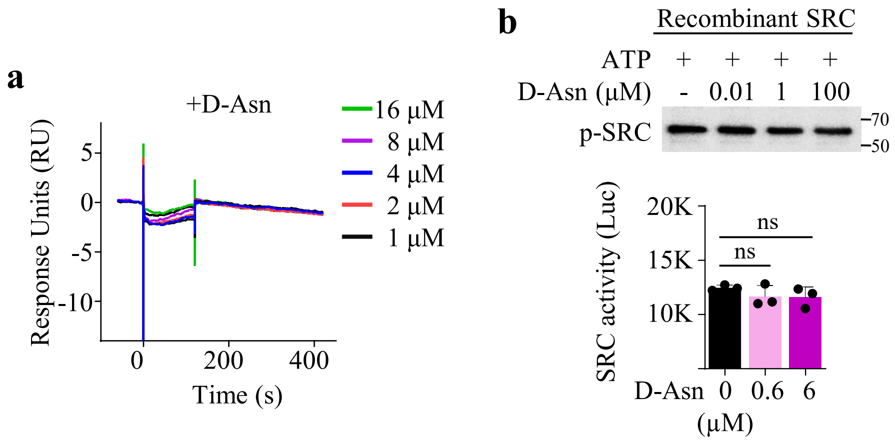

Fig. S12. D-Asparagine failed to interact with SRC. (a) BIAcore analysis revealed no binding of D-asparagine to recombinant SRC. **(b)** D-Asparagine had no effect on recombinant SRC phosphorylation and kinase activity (n=3). Each dot represents an independent sample. Data are presented as mean ± SD. Two-tailed Student's t-test for two group comparison **(b)**. ns, no significance. Source data are provided as a Source Data file.

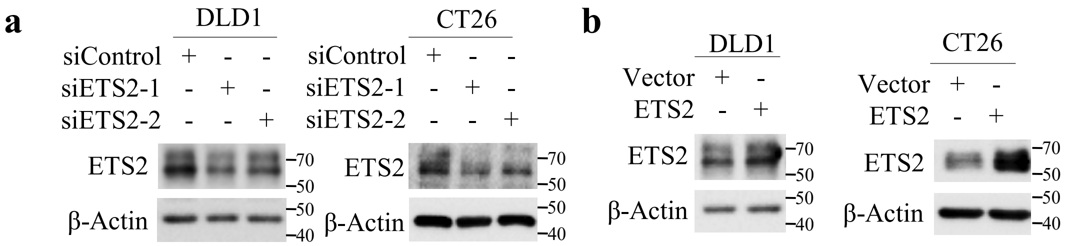

Fig. S13. Validation of ETS2 knockdown and overexpression in KRAS-mutant CRC cells. (a) siETS2 in DLD1 and CT26 cells. **(b)** Ectopic expression of ETS2 in DLD1 and CT26 cells. Experiments were repeated three times independently with similar results.

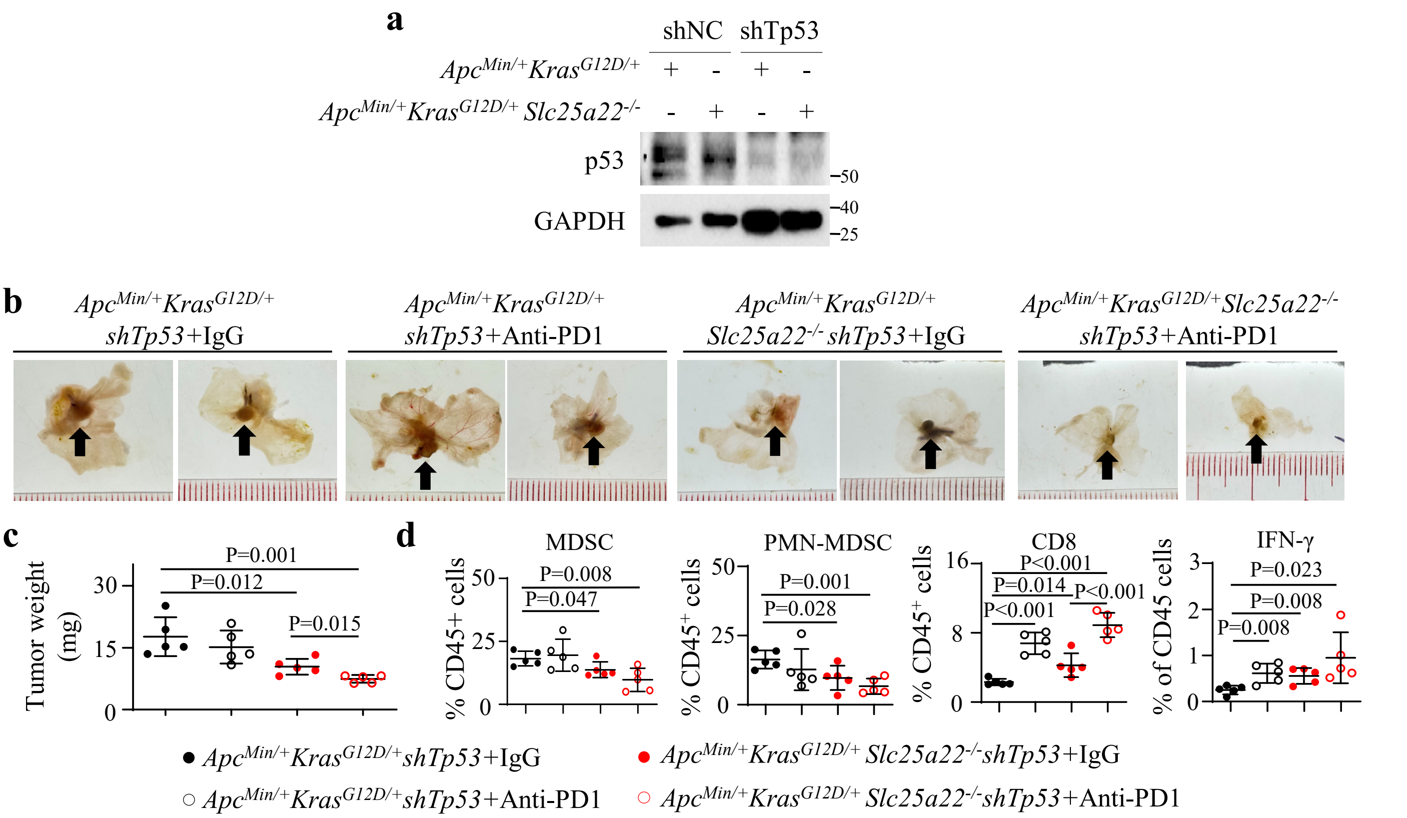

Fig. S14. SLC25A22 knockout plus anti-PD1 synergistically inhibited tumor growth in an orthotopic model of APC-KRAS organoids with TP53 knockdown. (a) shTp53 in *Apc*$^{Min/+}$*Kras*$^{G12D/+}$ and *Apc*$^{Min/+}$*Kras*$^{G12D/+}$ *Slc25a22*$^{-/-}$ organoids. **(b)** Representative images showing the orthotopic tumors from *Apc*$^{Min/+}$*Kras*$^{G12D/+}$*shTp53* and *Apc*$^{Min/+}$*Kras*$^{G12D/+}$ *Slc25a22*$^{-/-}$*shTp53* organoids implanted in C57BL/6 mice and treated with anti-PD1. **(c)** Tumor size in different groups (n=5). **(c)** Flow cytometry analysis of MDSC, PMN-MDSC, CD8$^+$ T-cells and IFN-γ$^+$ CD8$^+$ T-cells in different groups (n=5). Each dot represents an independent tumor. Data are presented as mean ± SD **(c, d).** Two-tailed Student's t-test for two group comparison **(c, d).** Source data are provided as a Source Data file.

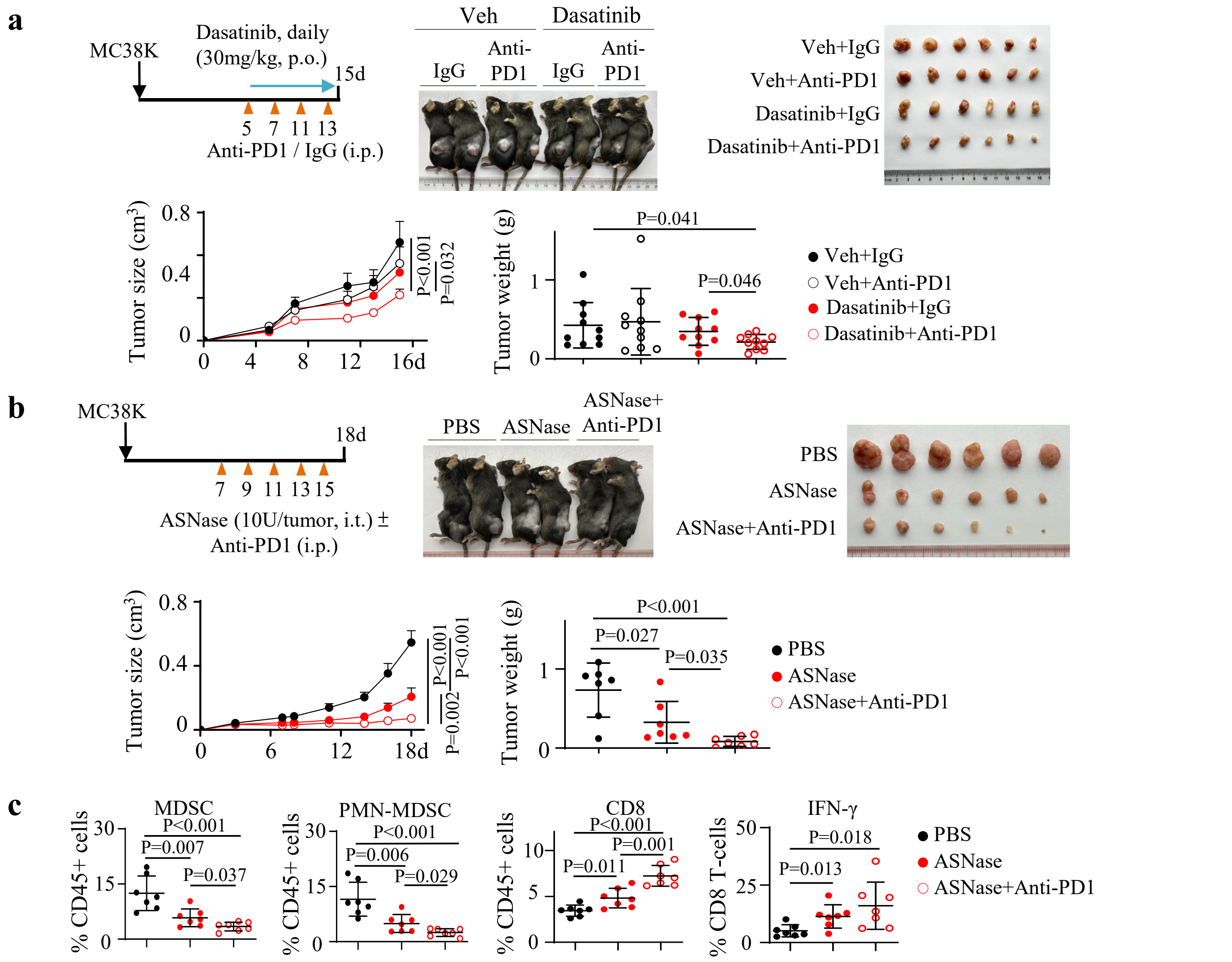

Fig. S15. SRC inhibitor sensitized KRAS-mutant CRC to anti-PD1 treatment. (a) Combination of Dasatinib plus anti-PD1 significantly inhibited growth of MC38K allografts, whilst single treatments were ineffective (n=10). **(b)** Combined Asparaginase plus anti-PD1 suppressed the growth of MC38K allografts, and was more effective than single treatment (n=7). **(c)** Flow cytometry analysis of MDSC, PMN-MDSC, $CD8^+$ T-cells and IFN-γ^+CD8^+ T-cells in different groups (n=7). Each dot represents an independent tumor. Data are presented as mean ± SEM for growth curve **(a, b)** and ± SD **(a-c)**. Two-tailed two-way ANOVA for growth curve comparison **(a-b)**. Two-tailed Student's t-test for two group comparison **(a-c).** Source data are provided as a Source Data file.

Table S1: Primers of Chip-PCR

Primer set	Sequence
#1-F:	CCAGTACCCCTGAGTAACCGA
#1-R:	CTGCTACCCAACTACCCCTAATG
#2-F:	ACAGGTCTTACCTATTGAGCTGG
#2-R:	GACATGGAGTAAGATGGGAGGAG
#3-F:	AGAGAGCTCTGAATCCCACTTG
#3-R	ACACACACATGTTGTTTTCTCTCA
#4-F:	TCTCTAGAAAGGGGGCCCAATA
#4-R	CGGATGAAATAGGAACGCCG
#5-F:	GCATGTCTGCTGCTTCAGTG
#5-R	GGCTCACCATTCCTGGTTCT
#6-F:	AGGGAAGAAGGAAGATAAGCAGG
#6-R	CCAGTGTTAGCGTCAGTGGA
#7-F:	CCACAGGAGTTACTCTGAAGGG
#7-R:	ATCCCGAGTCCGGAAGGA
Negative-F:	AATCCGAGACACAACGCTCTT
Negative-R:	GCCTGCGCTGAAGATACCA

Table S2: Antibody for flow cytometry

Specificities	Target	Brand	Fluorescein	Clone	Catalog number	Dilution
Human	CD45	Biolegend	BV605	HI30	304042	1:100
Human	CD3	Biolegend	PE	OKT3	317308	1:100
Human	CD4	Biolegend	FITC	A161A1	357405	1:100
Human	CD8	Biolegend	PE/Cyanine5	SK1	344770	1:100
Human	MHC-II	Biolegend	PE	LN3	327007	1:100
Human	CD11b	Biolegend	FITC	ICRF44	301330	1:100
Human	CD33	Biolegend	PE/Cyanine7	P67.6	366618	1:100
Mouse	CD45	Biolegend	BV605	30-F11	103140	1:100
Mouse	CD3	Biolegend	PE	17A2	100206	1:100
Mouse	CD4	Biolegend	FITC	RM4-5	100509	1:100
Mouse	CD8	Biolegend	BV421	53-6.7	100738	1:100
Mouse	IFN-γ	Biolegend	FITC	XMG1.2	505806	1:100
Mouse	TNF-α	Biolegend	PE/Cyanine7	MP6-XT22	506324	1:100
Mouse	Granzyme B	Biolegend	PerCP/Cyanine5.5	QA16A02	372212	1:100
Mouse	CD11b	Biolegend	PerCP/Cyanine5.5	M1/70	101228	1:100
Mouse	Ly-6G/Ly-6C	Biolegend	FITC	RB6-8C5	108406	1:100
Mouse	Ly-6G	Biolegend	PE/Cyanine7	1A8	127618	1:100
Mouse	Ly-6C	Biolegend	PE	HK1.4	128008	1:100
Mouse	CD274	Biolegend	BV711	10F.9G2	124319	1:100
Mouse	CD11c	Biolegend	PE	N418	117308	1:100
Mouse	CD206	Biolegend	BV421	C068C2	141717	1:100
Mouse	F4/80	Biolegend	FITC	BM8	123108	1:100

Table S3: Primers for RT-qPCR.

Species	Gene	sequence
Human	CXCL1-F	AGCTTGCCTCAATCCTGCATCC
Human	CXCL1-R	TCCTTCAGGAACAGCCACCAGT
Human	CXCL3-F	TTCACCTCAAGAACATCCAAAGTG
Human	CXCL3-R	TTCTTCCCATTCTTGAGTGTGGC
Human	ETS2-F	CCCCTCGGTCGTGCG
Human	ETS2-R	CAGCAAACAGGGACCCATCAA
Human	CEBPB-F	GCAACCCACGTGTAACTGTC
Human	CEBPB-R	GCCCCCAAAAGGCTTTGTAAC
Human	JunB-F	AAGGGACACGCCTTCTGAAC
Human	JunB-R	AAACGTCGAGGTGGAAGGAC
Human	JunD-F	ATGATGAAGAAGGACGCGCT
Human	JunD-R	TTGGACTGGATGATGAGGCG
Human	beta-actin-F	CTCACCATGGATGATGATATCGC
Human	beta-actin-R	GGAATCCTTCTGACCCATGCC
Mouse	CXCL1-F	TCCAGAGCTTGAAGGTGTTGCC
Mouse	CXCL1-R	AACCAAGGGAGCTTCAGGGTCA
Mouse	CXCL3-F	TGAGACCATCCAGAGCTTGACG
Mouse	CXCL3-R	CCTTGGGGGTTGAGGCAAACTT
Mouse	ETS2-F	ATGCTGTGTAACCTCGGCAA
Mouse	ETS2-R	CTGTTCCATGCTGAAGCCTAATG
Mouse	CEBPB-F	GCTGAGCGACGAGTACAAGAT
Mouse	CEBPB-R	CAGCTGCTTGAACAAGTTCCG
Mouse	ETS2-F	ATGCTGTGTAACCTCGGCAA
Mouse	ETS2-R	CTGTTCCATGCTGAAGCCTAATG
Mouse	JunB-F	GTCTCCTACGGGAGCAAGTG
Mouse	JunB-R	GGAGTCCAGTGTGTGAGCTG
Mouse	JunD-F	TACGCAGTTCCTCTACCCGA
Mouse	JunD-R	AAACTGCTCAGGTTGGCGTA
Mouse	Arg1-F	CCTTTCTCAAAAGGACAGCCTC
Mouse	Arg1-R	CAGACCGTGGGTTCTTCACA
Mouse	INOS-F	TCTAGTGAAGCAAAGCCCAACA
Mouse	INOS-R	CTCTCCACTGCCCCAGTTTT
Mouse	PDL1-F	CCTCGCCTGCAGATAGTTCC
Mouse	PDL1-R	CCCAGTACACCACTAACGCA
Mouse	beta-actin-F	GGTACCACCATGTACCCAGG
Mouse	beta-actin-R	AAAACGCAGCTCAGTAACAGTC

Table S4: The sequence of sgRNA targeting SLC25A22

Species	Gene	sequence
Mouse	SLC25A22	sgRNA-1: ATACATGCCGAAGTAGCCCT
Mouse	SLC25A22	sgRNA-2: CCCGGAGAAGGCCATCAAGT

Table S5: Antibody for Western blot

Target	Brand	Catalog number
SLC25A22	Sigma-Aldrich	HPA014662 (1:2000)
ETS2	Invitrogen	PA5-28053 (1:1000)
Phospho-ETS2	Invitrogen	44-1105G (1:700)
Lamin A/C	Cell signaling	4777 (1:3000)
ASNS	Abclonal	A1030 (1:1000)
Anti-Src antibody [GD11]	Abcam	ab231081 (1:5000)
Anti-Src (phospho Y419) antibody [EPR17734]	Abcam	ab185617 (1:1000)
p44/42 MAPK (Erk1/2)	Cell signaling	9102 (1:1000)
Phospho-p44/42 MAPK (Erk1/2) (Thr202/Tyr204)	Cell signaling	9101 (1:1000)
GAPDH	Cell signaling	5174 (1:2000)
β-actin	Cell signaling	4970 (1:2000)

Table S6: siRNA

Gene	Species	Brand	Catalog number
siCXCL1-1	Human	Invitrogen	s6215
siCXCL1-2	Human	Invitrogen	s6216
siCXCL3-1	Human	Invitrogen	s6221
siCXCL3-2	Human	Invitrogen	s6223
siASNS-1	Human	Invitrogen	s533751
siASNS-2	Human	Invitrogen	s533753
siETS2-1	Human	Invitrogen	115625
siETS2-2	Human	Invitrogen	146636
siCXCL1-1	Mouse	Invitrogen	s67077
siCXCL1-2	Mouse	Invitrogen	s67078
siASNS-1	Mouse	Invitrogen	s77506
siASNS-2	Mouse	Invitrogen	s77504
siETS2-1	Mouse	Invitrogen	187766
siETS2-2	Mouse	Invitrogen	187767
