# Supplementary material for: Targeting of SLC25A22 boosts the immunotherapeutic response in KRAS-mutant colorectal cancer
Source: Nat Commun. 2023 Aug 4;14:4677. doi: 10.1038/s41467-023-39571-6 (PMC10403583; doi:10.1038/s41467-023-39571-6)
Supplement: Supplementary file 4 — Source Data [file 41467_2023_39571_MOESM4_ESM.zip › 230525_Source Data-uncropped blots and gels.pdf]

Figure 1F

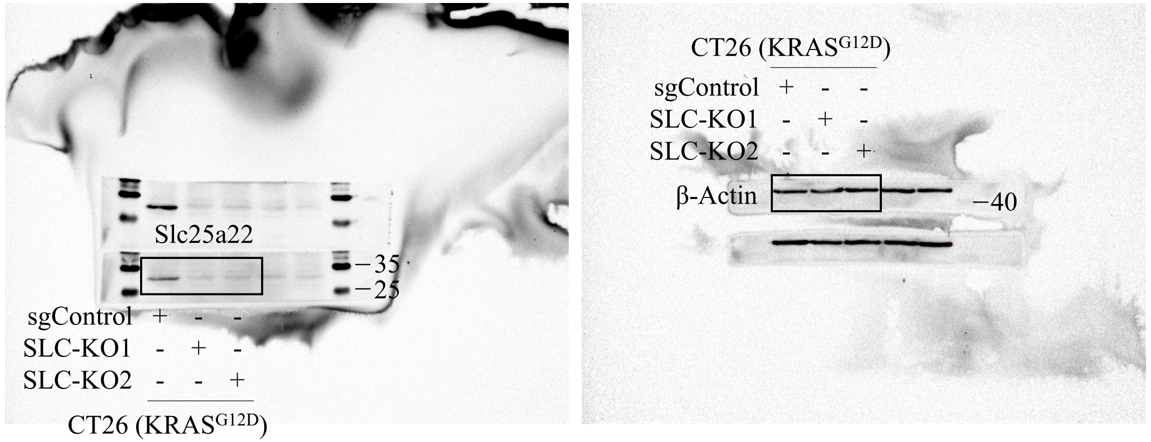
Slc25a22
−35
−25
sgControl + - -
SLC-KO1 - + -
SLC-KO2 - - +
CT26 (KRAS^{G12D})
CT26 (KRAS^{G12D})
sgControl + - -
SLC-KO1 - + -
SLC-KO2 - - +
β-Actin
−40

Figure 6I

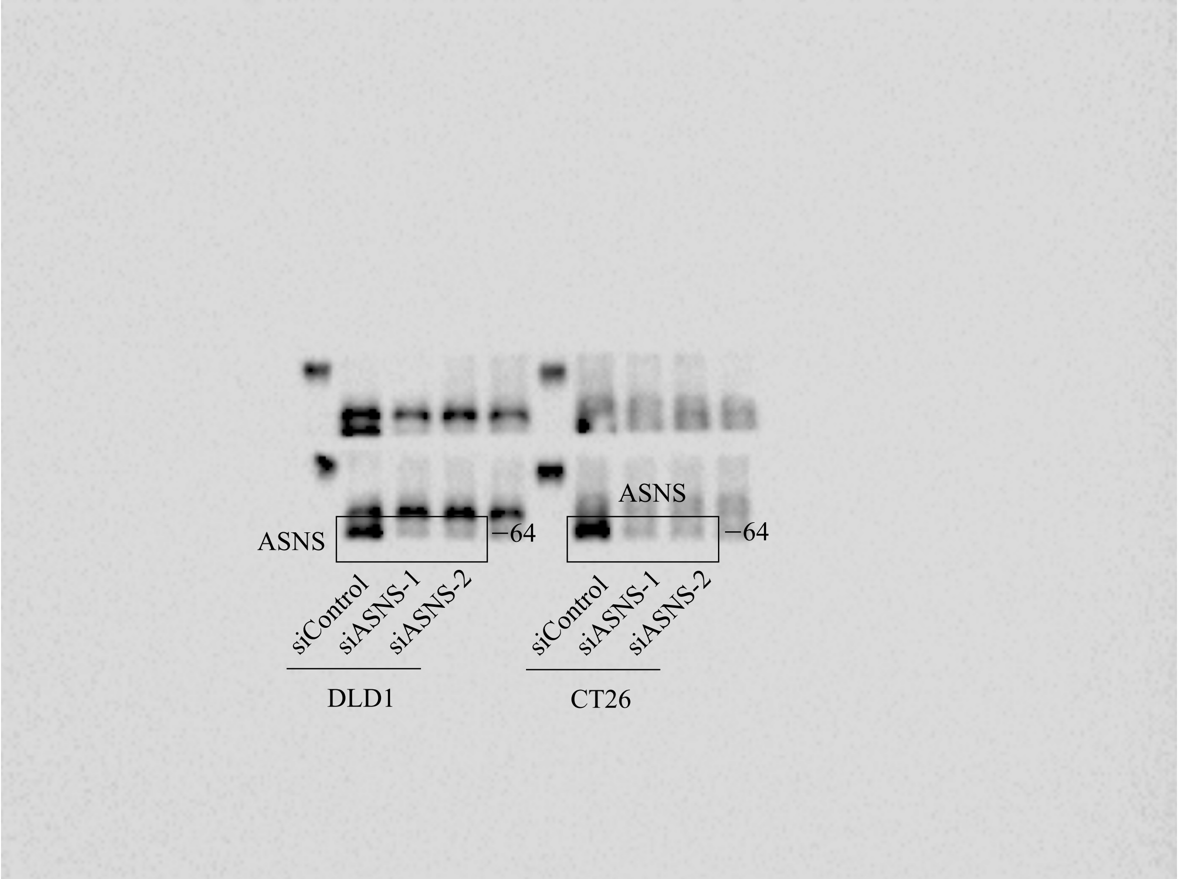

ASNS
ASNS
–64
–64
siControl
siASNS-1
siASNS-2
siControl
siASNS-1
siASNS-2
DLD1
CT26

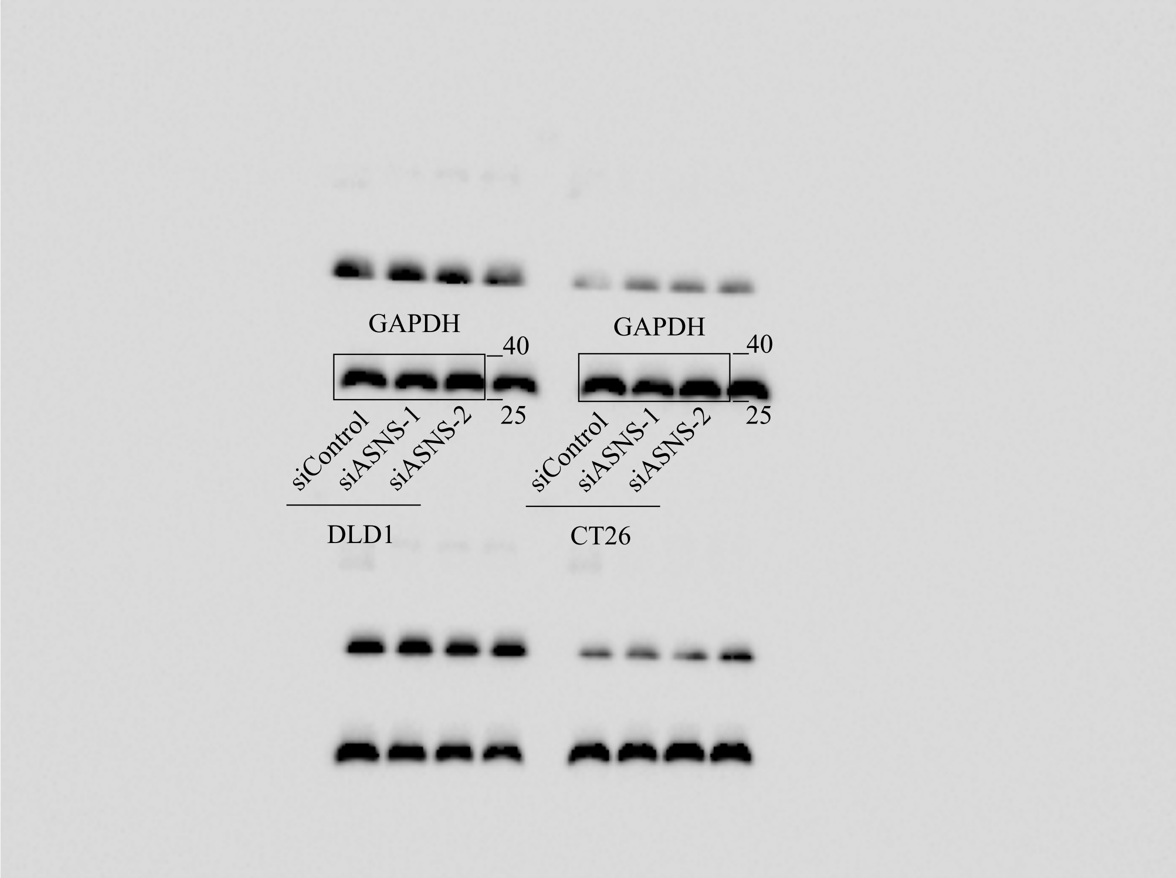

GAPDH
GAPDH
40
25
40
25
siControl
siASNS-1
siASNS-2
siControl
siASNS-1
siASNS-2
DLD1
CT26

Figure 7A

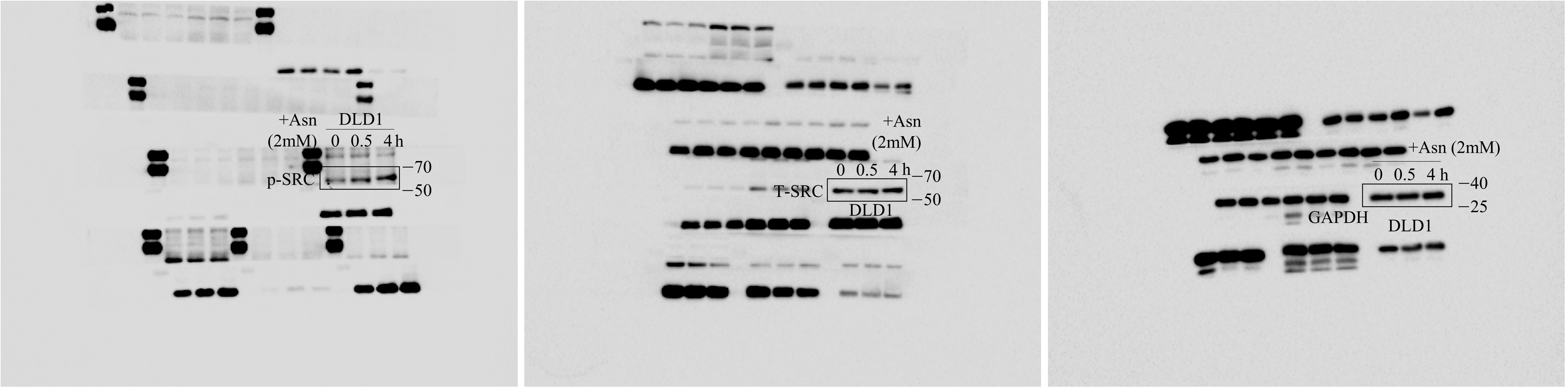

+Asn
(2mM)
DLD1
0 0.5 4 h
p-SRC
70
50
+Asn
(2mM)
0 0.5 4 h
T-SRC
70
50
DLD1
+Asn (2mM)
0 0.5 4 h
40
25
GAPDH
DLD1

Figure 7B DLD1

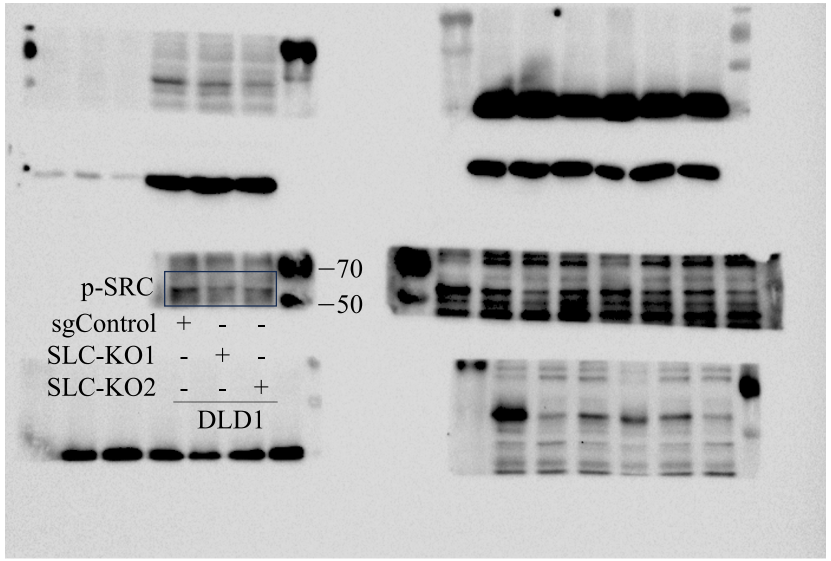

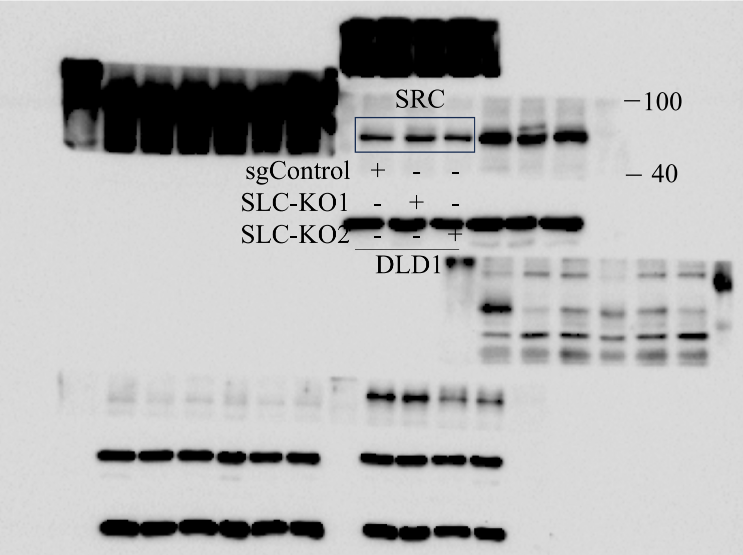

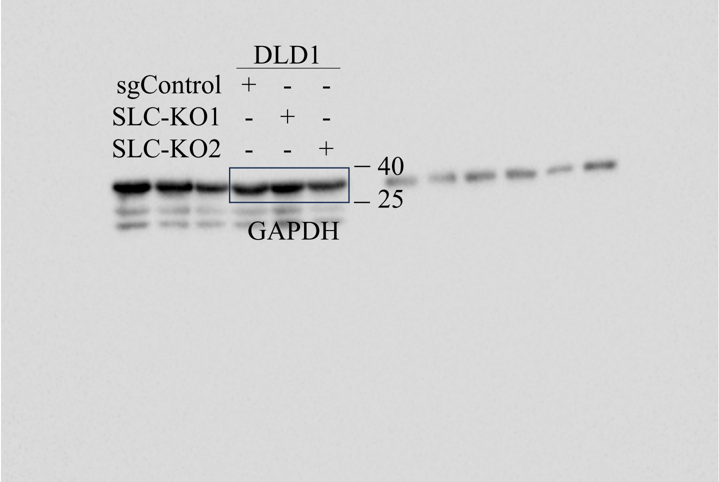

Figure 7B CT26

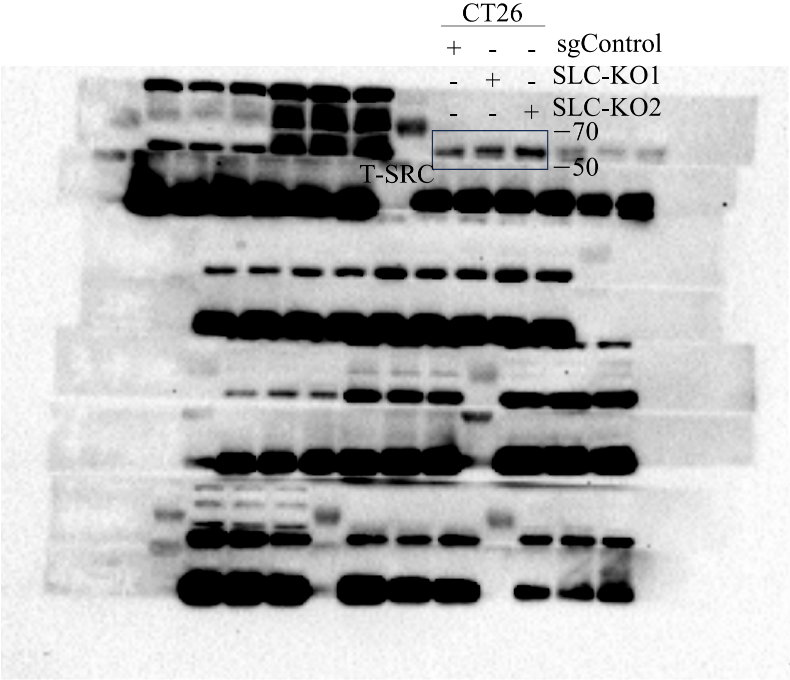

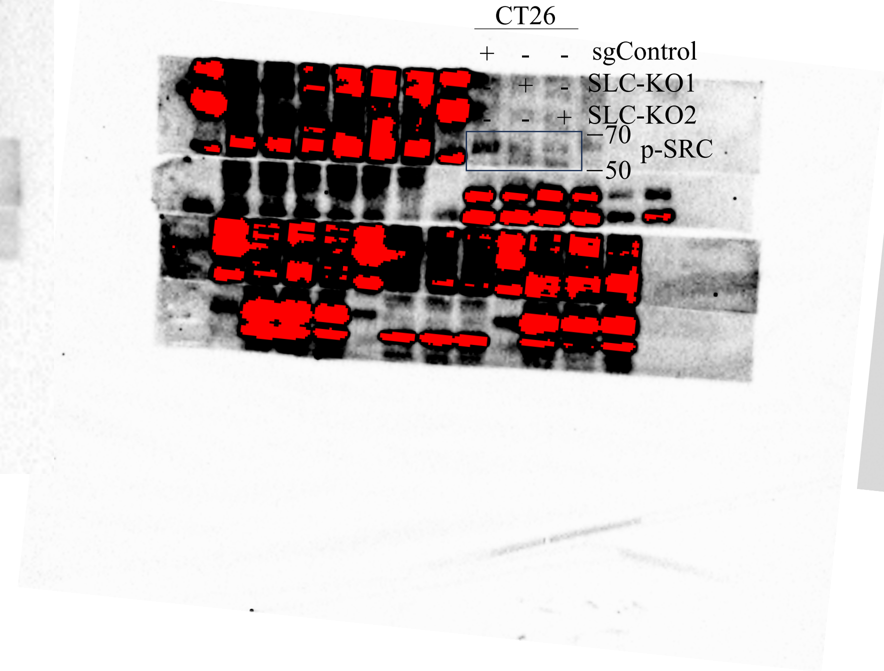

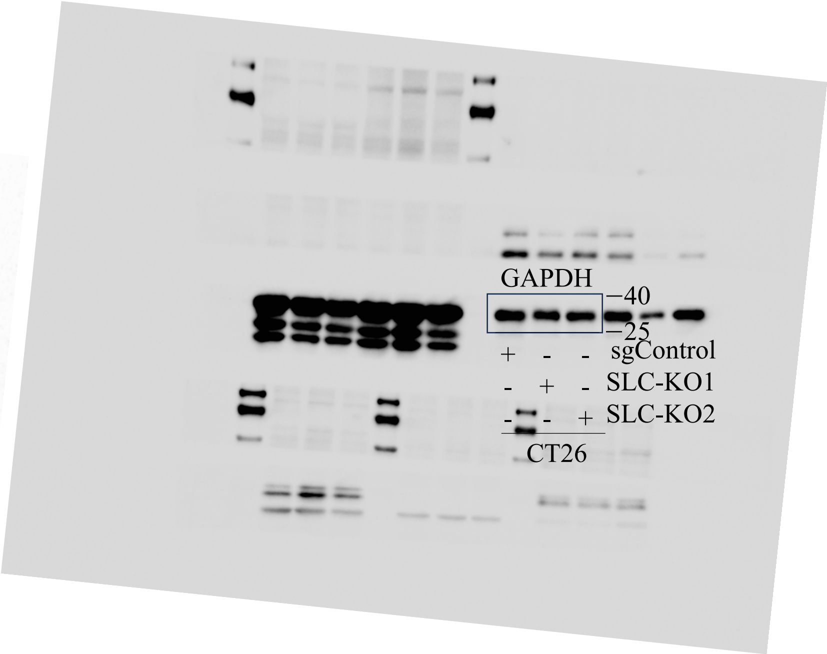

Figure 7C

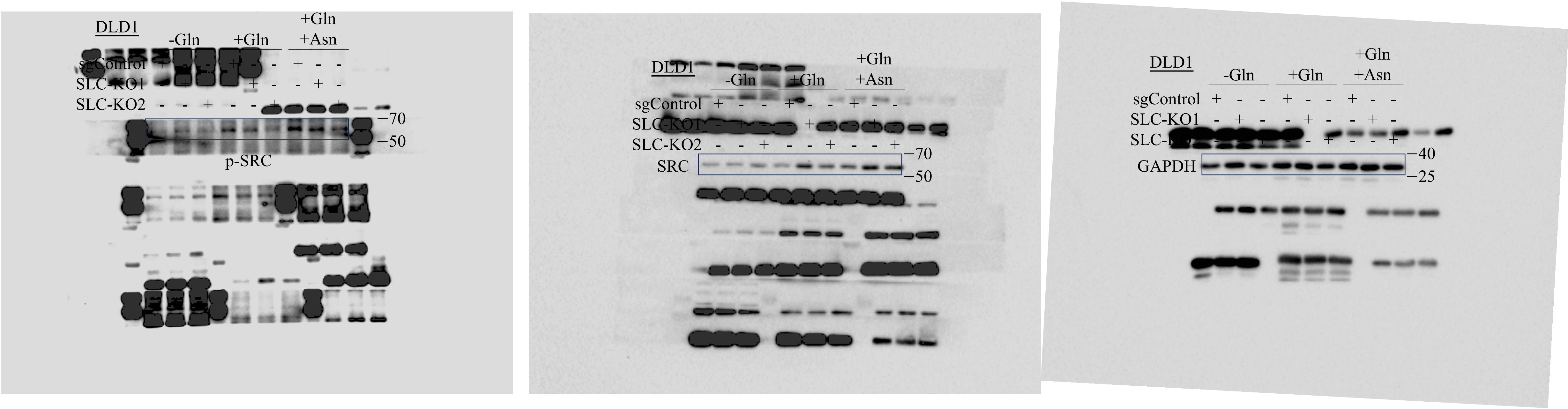

DLD1
-Gln
+Gln
+Gln +Asn
sgControl
SLC-KO1
SLC-KO2
70
50
p-SRC
SRC
40
25
GAPDH

Figure 7D

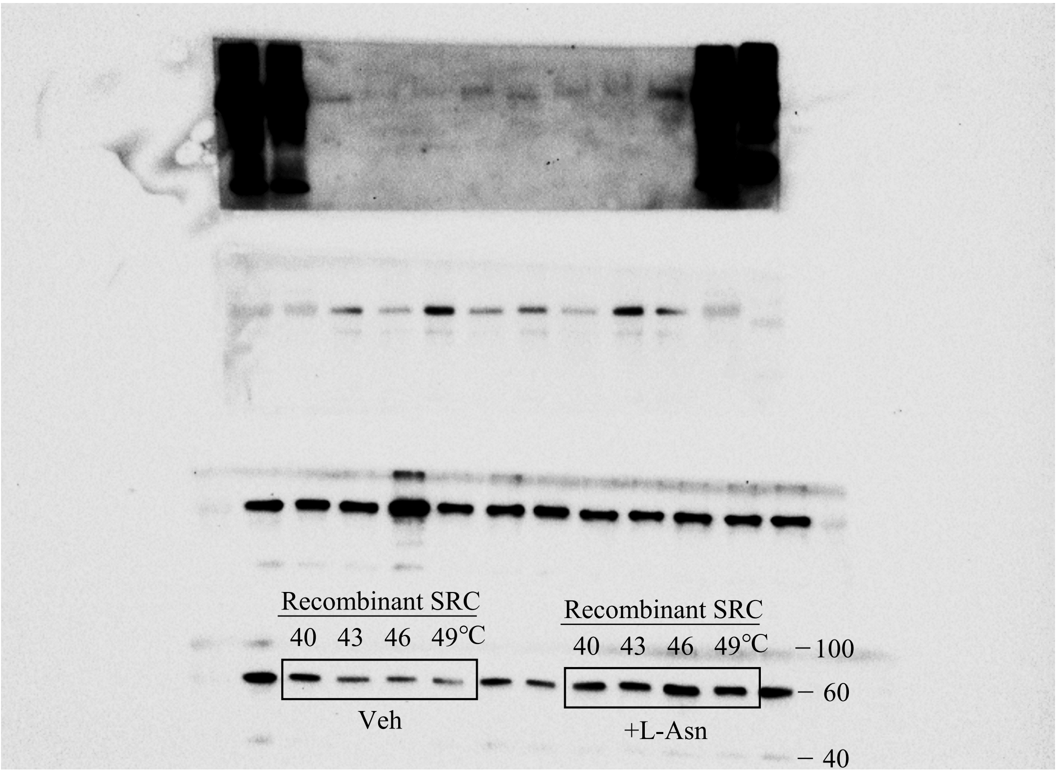
Recombinant SRC
40 43 46 49°C
Veh
Recombinant SRC
40 43 46 49°C
+L-Asn
100
60
40

Figure 7F

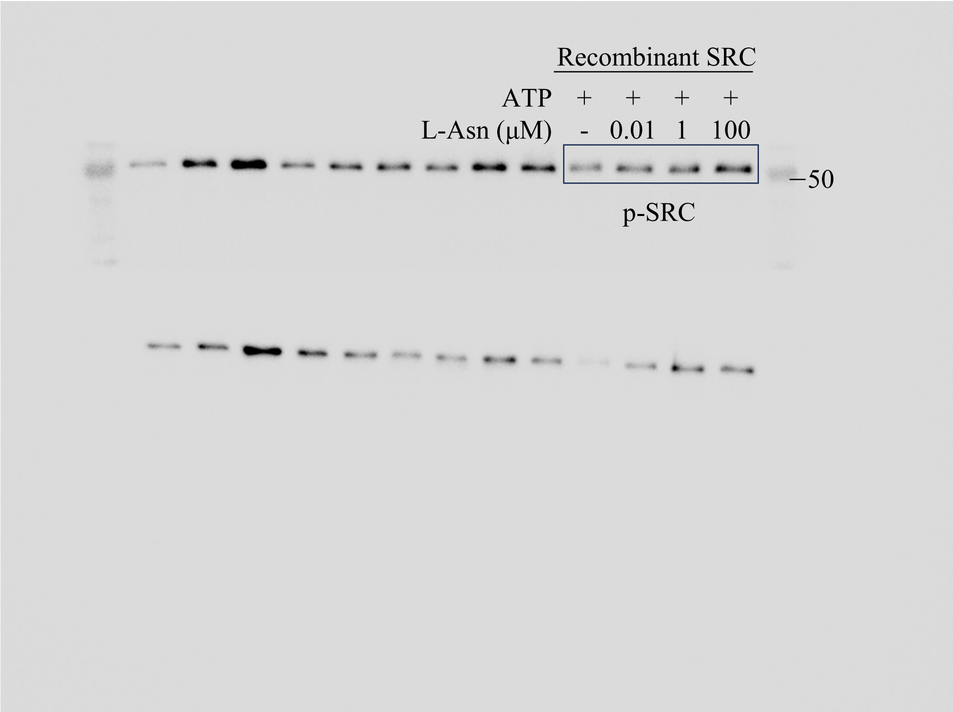

Figure 7H DLD1

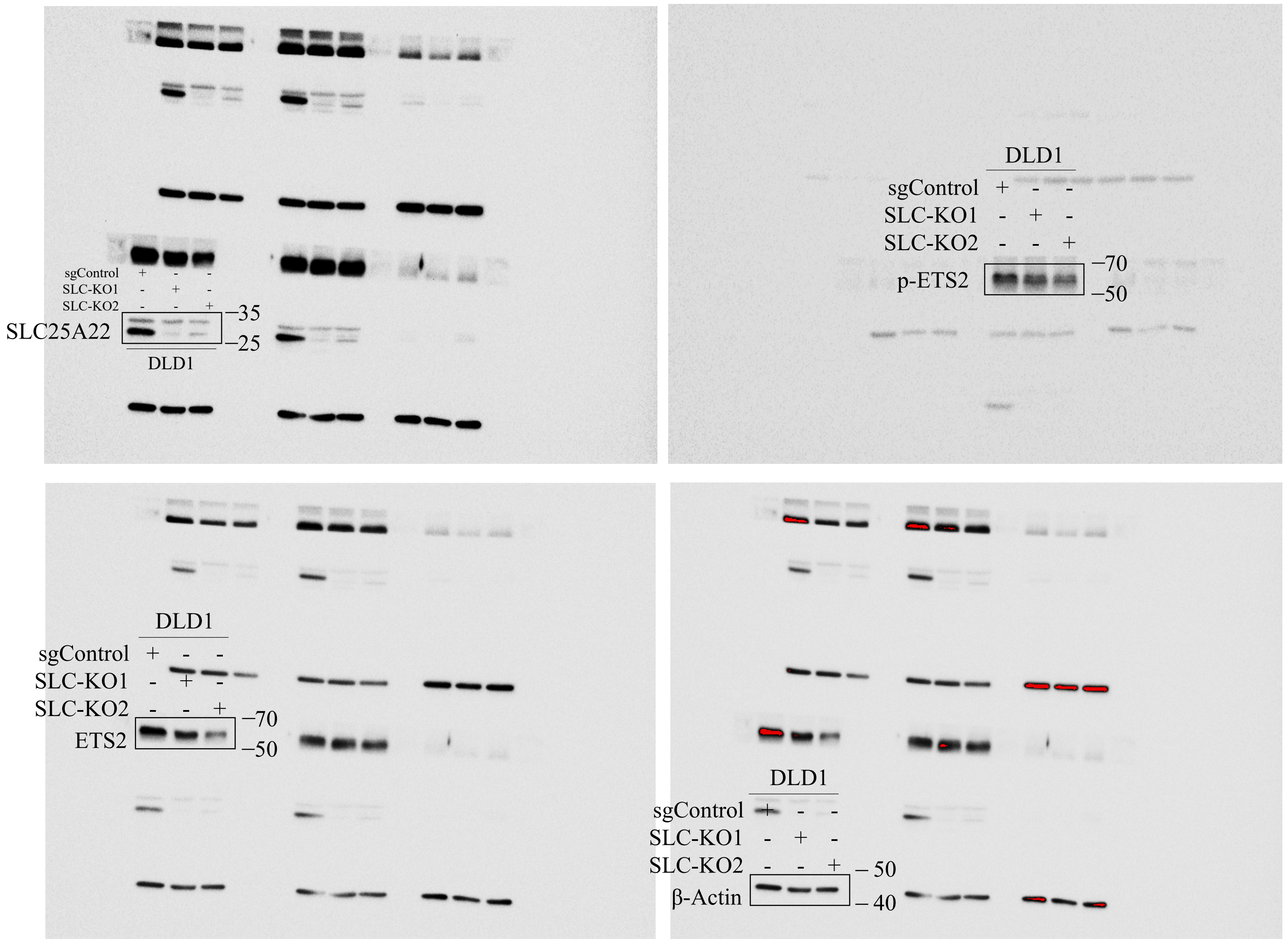

Figure 7H CT26

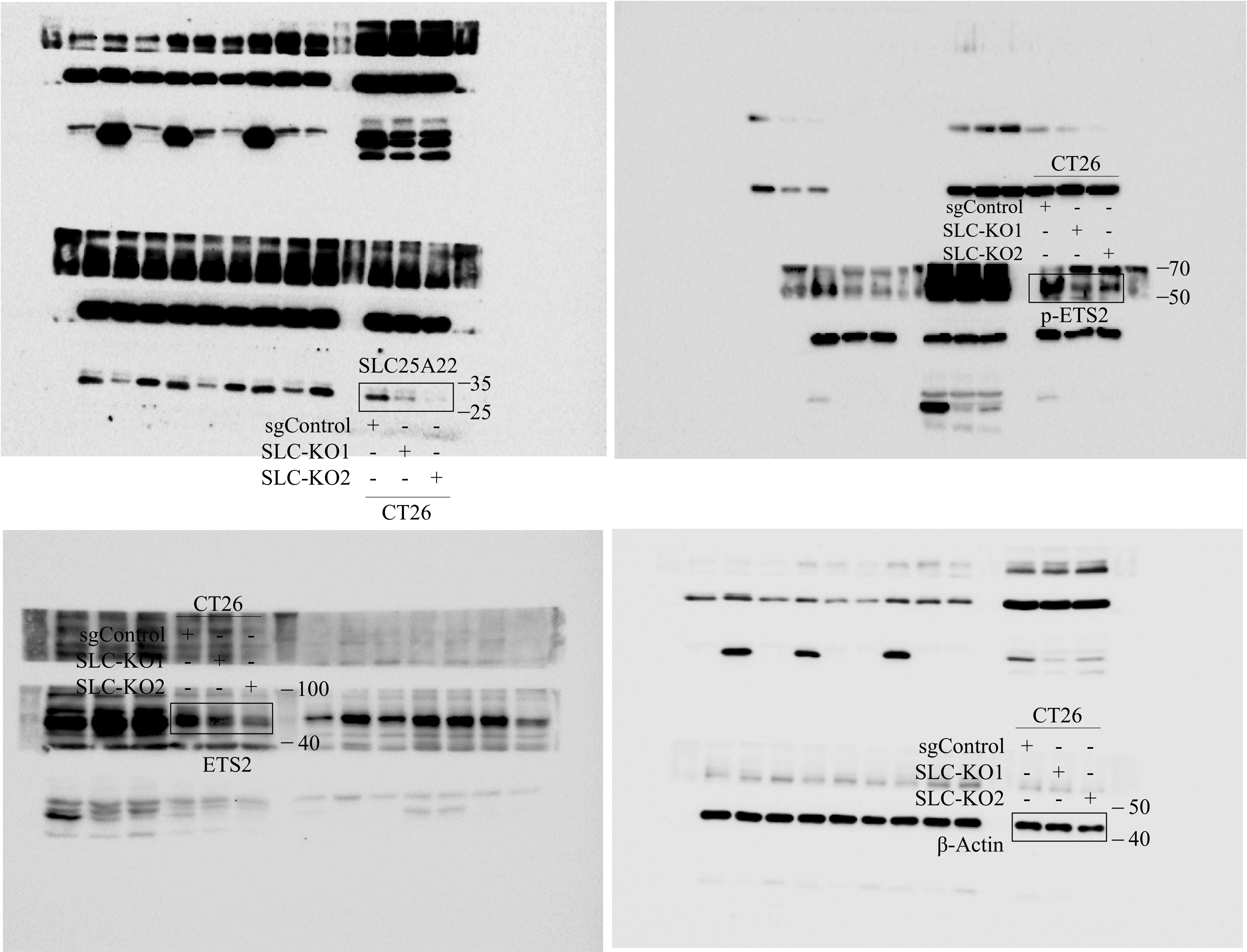

Figure 7I

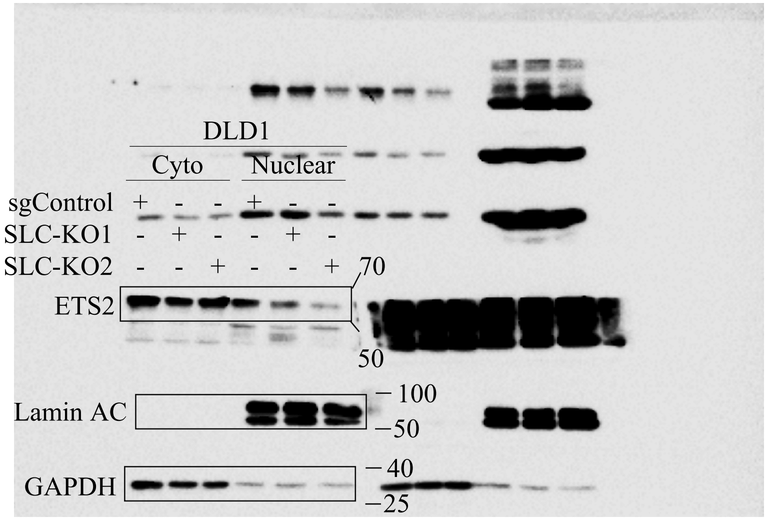
DLD1
Cyto
Nuclear
sgControl + - - + - -
SLC-KO1 - + - - + -
SLC-KO2 - - + - - +
70
ETS2
50
Lamin AC
100
50
GAPDH
40
25

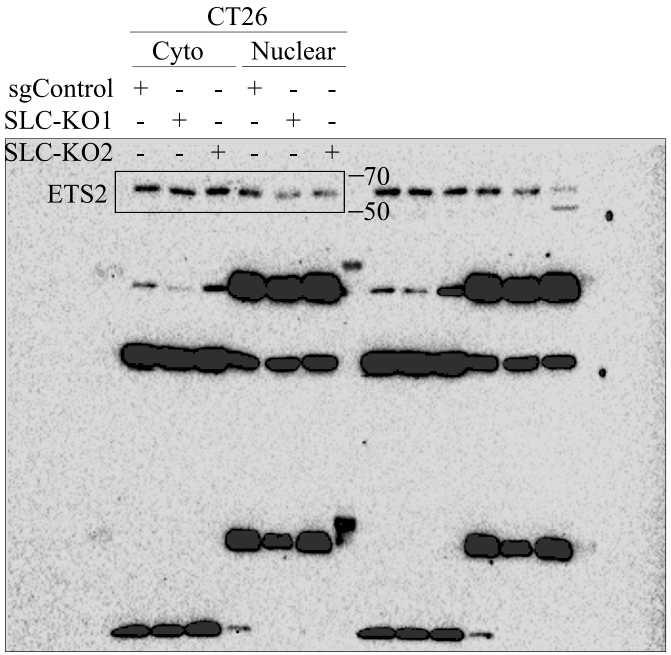
CT26
Cyto
Nuclear
sgControl + - - + - -
SLC-KO1 - + - - + -
SLC-KO2 - - + - - +
ETS2
70
50

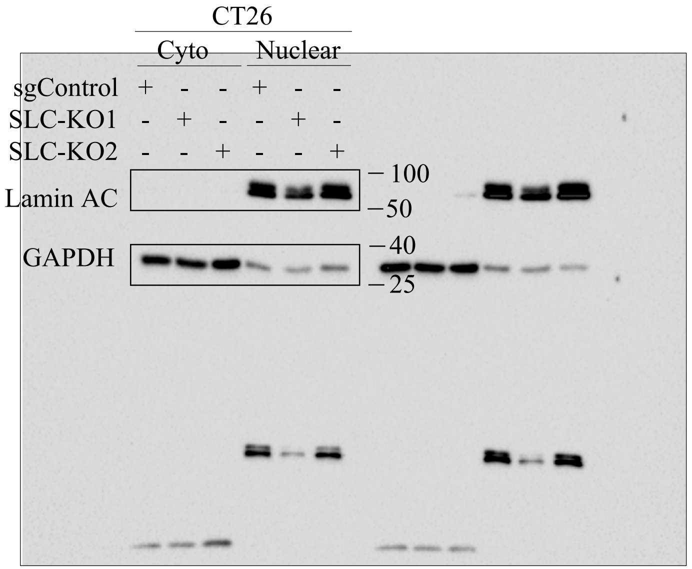
CT26
Cyto
Nuclear
sgControl + - - + - -
SLC-KO1 - + - - + -
SLC-KO2 - - + - - +
Lamin AC
100
50
GAPDH
40
25

Figure 7J

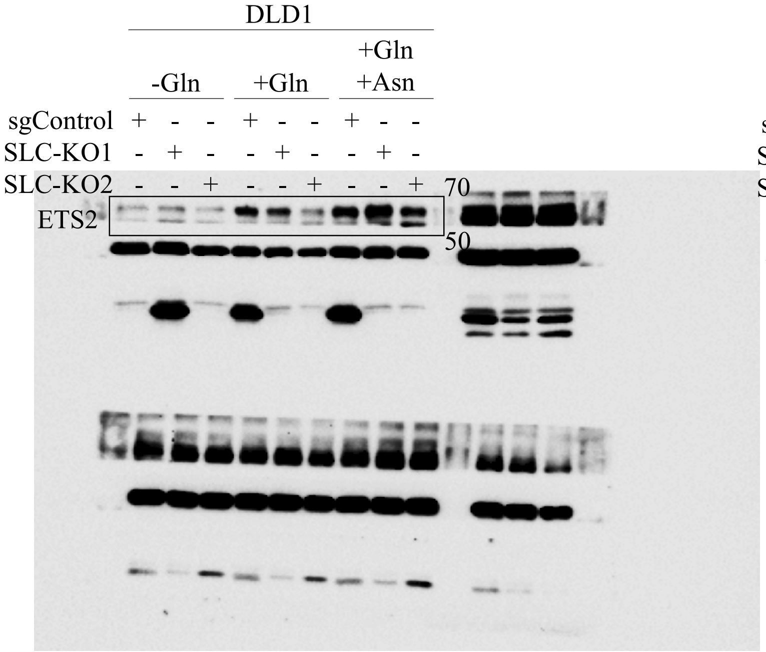
DLD1
-Gln
+Gln
+Gln
+Asn
sgControl + - - + - - + - -
SLC-KO1 - + - - + - - + -
SLC-KO2 - - + - - + - - +
70
ETS2
50

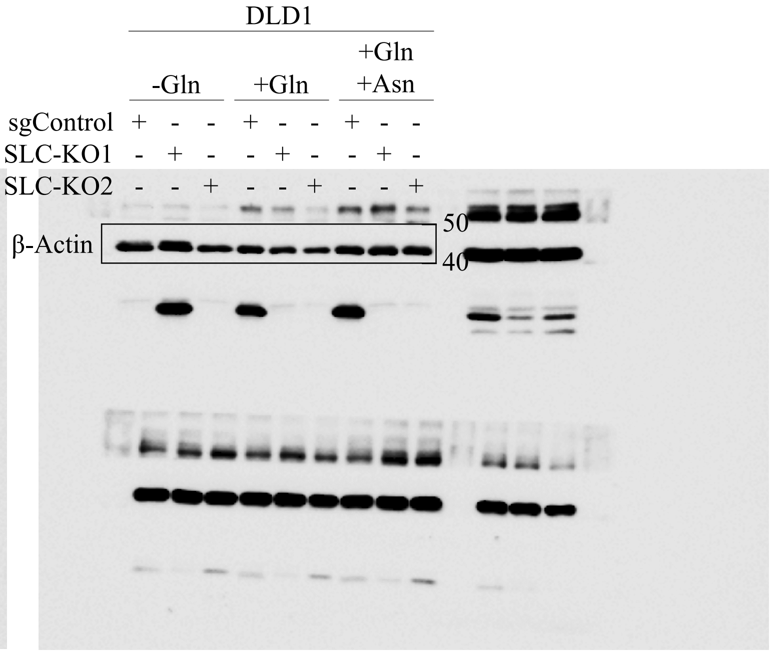
DLD1
-Gln
+Gln
+Gln
+Asn
sgControl + - - + - - + - -
SLC-KO1 - + - - + - - + -
SLC-KO2 - - + - - + - - +
50
β-Actin
40

Figure 7K

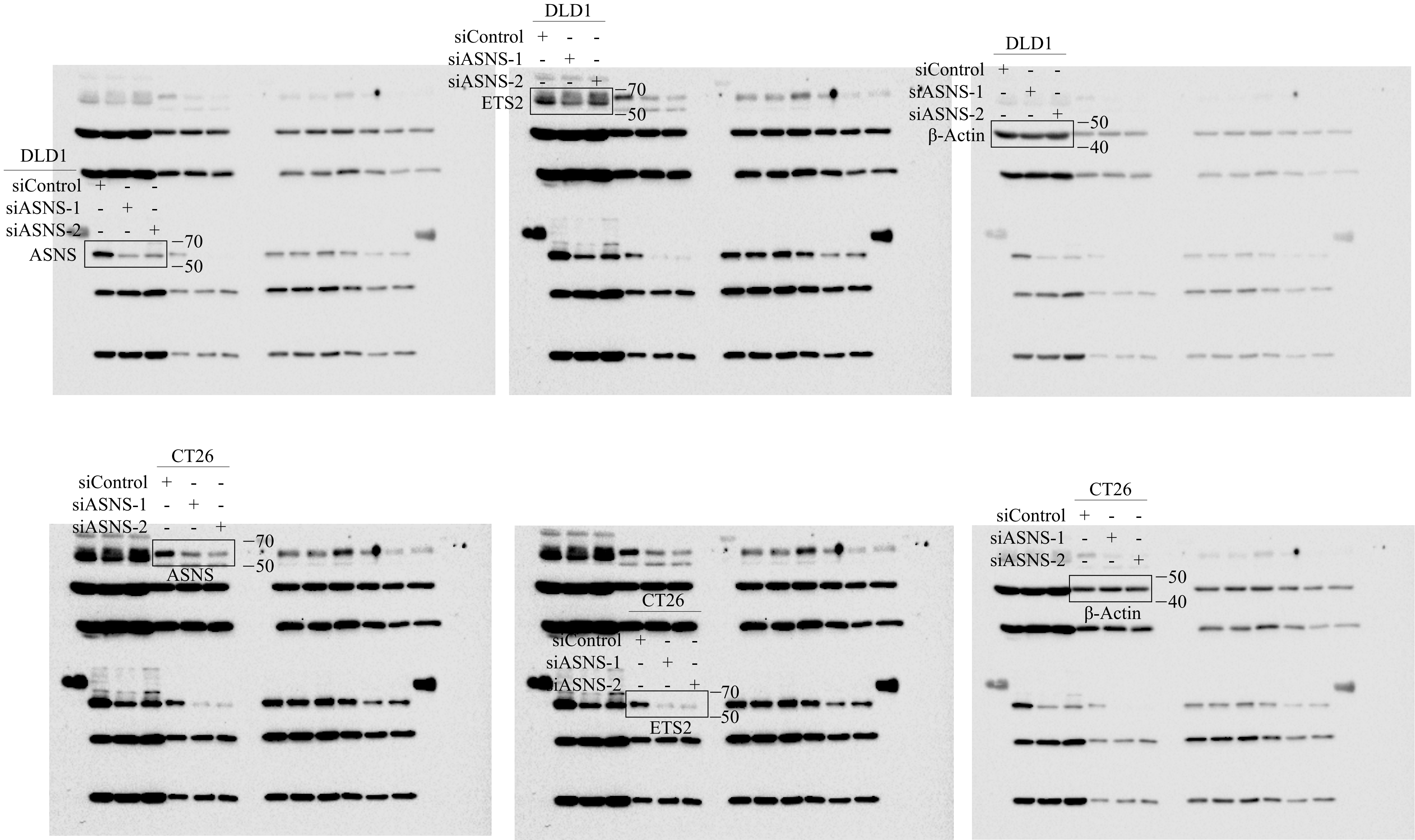
DLD1
siControl + - -
siASNS-1 - + -
siASNS-2 - - +
ASNS
70
50
DLD1
siControl + - -
siASNS-1 - + -
siASNS-2 - - +
ETS2
70
50
DLD1
siControl + - -
siASNS-1 - + -
siASNS-2 - - +
β-Actin
50
40
CT26
siControl + - -
siASNS-1 - + -
siASNS-2 - - +
ASNS
70
50
CT26
siControl + - -
siASNS-1 - + -
siASNS-2 - - +
ETS2
70
50
CT26
siControl + - -
siASNS-1 - + -
siASNS-2 - - +
β-Actin
50
40

Figure 7P

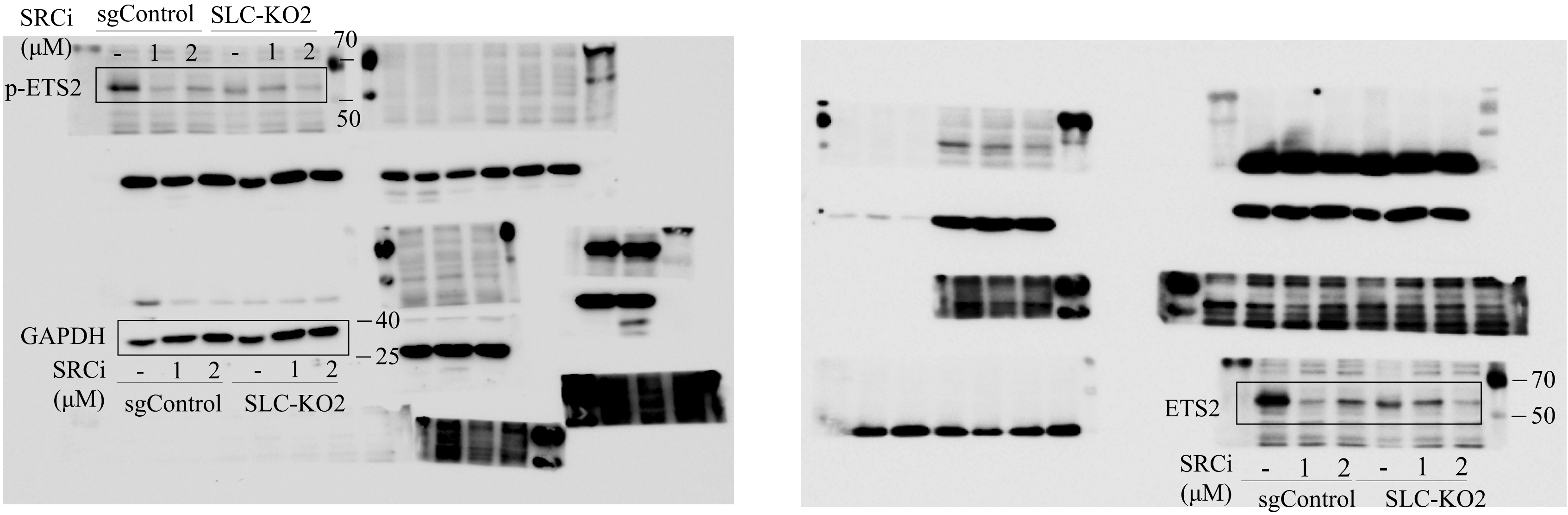

SRCi (μM)
sgControl
SLC-KO2
- 1 2 - 1 2
p-ETS2
70
50
GAPDH
40
25
SRCi (μM)
- 1 2 - 1 2
sgControl
SLC-KO2
ETS2
70
50
SRCi (μM)
- 1 2 - 1 2
sgControl
SLC-KO2

Figure S12B

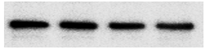

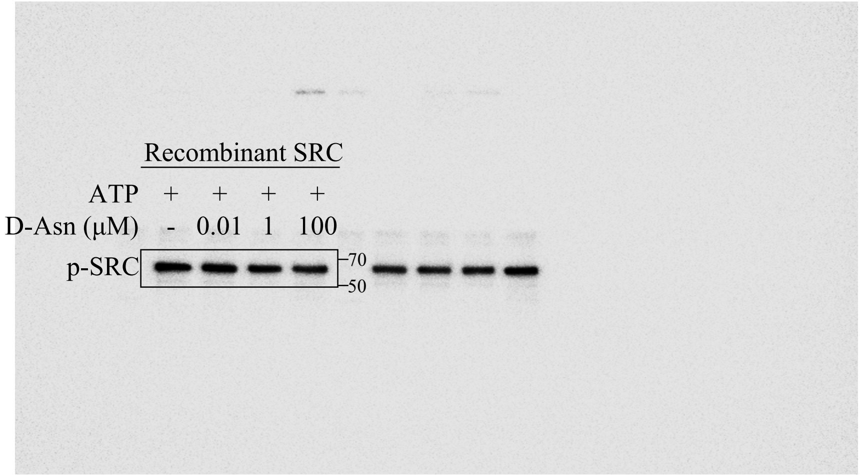

Figure S13A

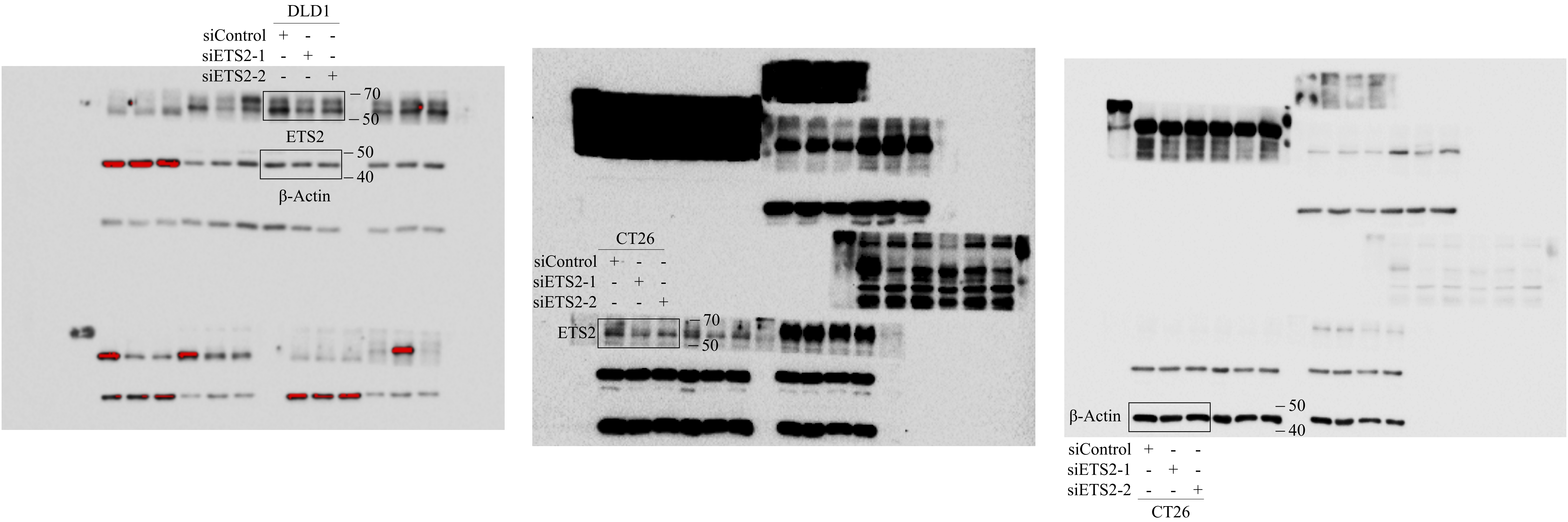

DLD1
siControl + - -
siETS2-1 - + -
siETS2-2 - - +
70
50
ETS2
50
40
β-Actin
CT26
siControl + - -
siETS2-1 - + -
siETS2-2 - - +
ETS2
70
50
β-Actin
50
40
siControl + - -
siETS2-1 - + -
siETS2-2 - - +
CT26

Figure S13B

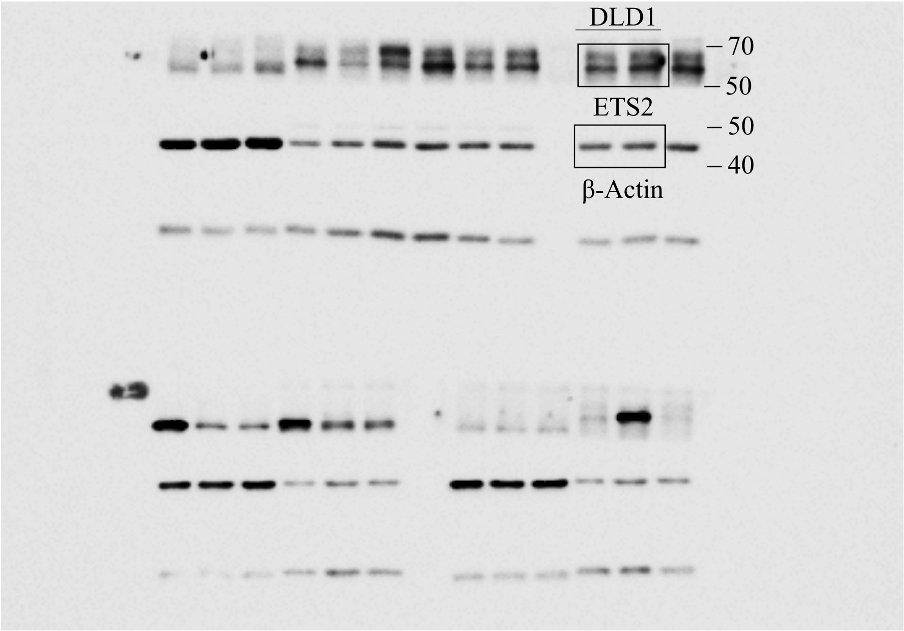
DLD1
70
50
ETS2
50
40
β-Actin

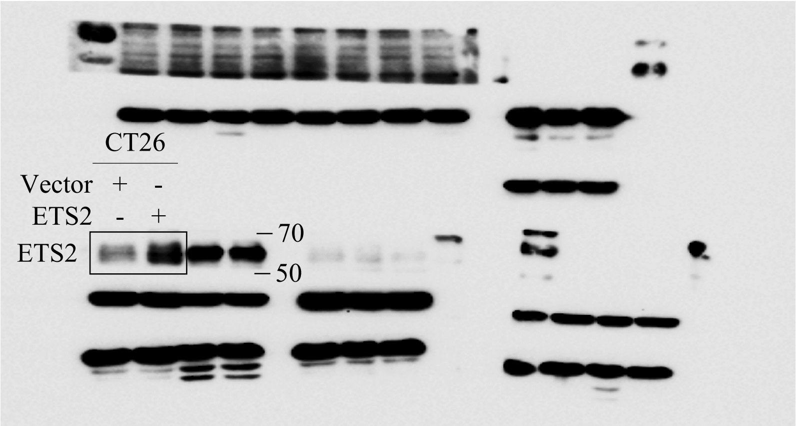
CT26
Vector + -
ETS2 - +
ETS2
70
50

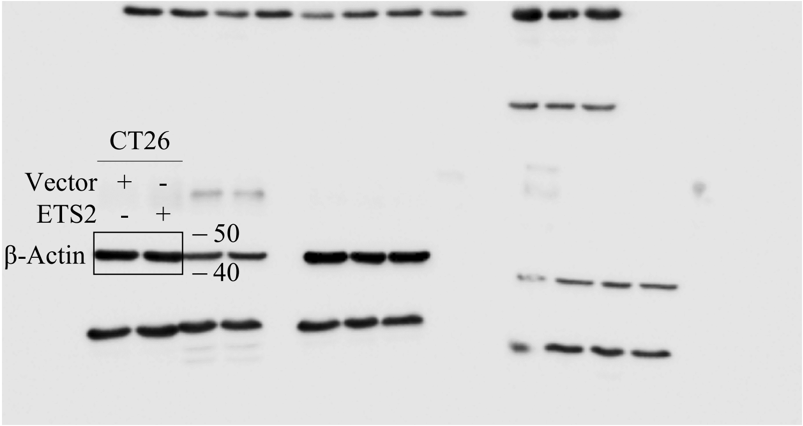
CT26
Vector + -
ETS2 - +
β-Actin
50
40

Figure S14A

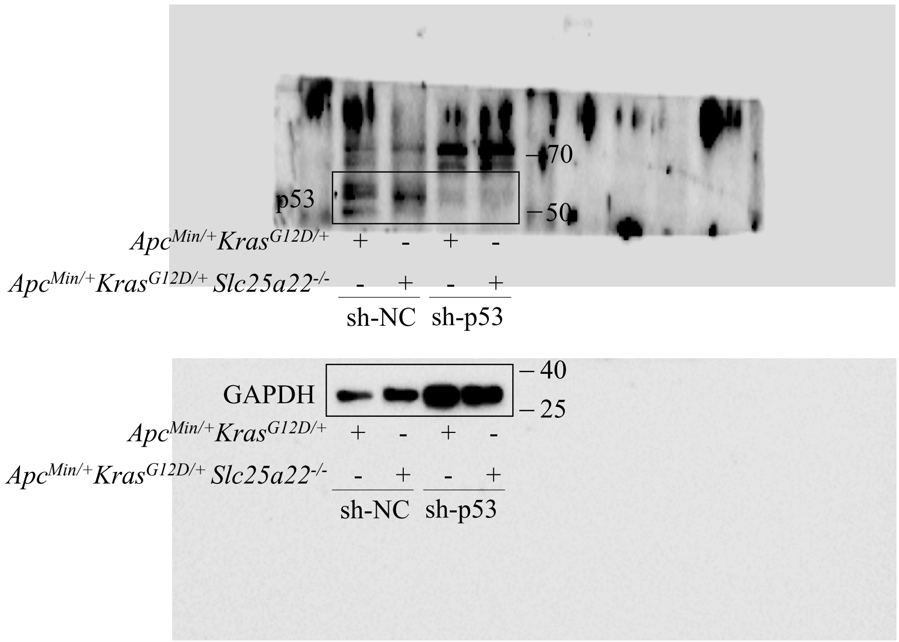

p53
70
50
ApcMin/+KrasG12D/+ + - + -
ApcMin/+KrasG12D/+ Slc25a22-/- - + - +
sh-NC sh-p53
GAPDH
40
25
ApcMin/+KrasG12D/+ + - + -
ApcMin/+KrasG12D/+ Slc25a22-/- - + - +
sh-NC sh-p53
